# Occupational Therapy's Unique Contribution to Chronic Pain Management: A Scoping Review

**DOI:** 10.1155/2018/5378451

**Published:** 2018-11-12

**Authors:** Émilie Lagueux, Andréa Dépelteau, Julie Masse

**Affiliations:** ^1^Research Center of the Centre Hospitalier Universitaire de Sherbrooke, Sherbrooke, Québec, Canada; ^2^Faculty of Medicine and Health Sciences, School of Rehabilitation, Université de Sherbrooke, Sherbrooke, Québec, Canada; ^3^Faculty of Medicine, School of Rehabilitation, Université de Montréal, Montréal, Québec, Canada

## Abstract

Occupational therapy (OT) makes a unique contribution to chronic pain (CP) management due to its overarching focus on occupation. The aim of this scoping review was to describe current knowledge about this contribution by documenting OT roles, models, assessments, and intervention methods used with adults living with CP. A systematic search exploring 10 databases and gray literature from 2006 to 2017 was conducted. Fifty-two sources were retained and analysed. Results bring forward the main role of OT being improving activities and participation (76.9 %), the Canadian Model of Occupational Performance (9.6 %), and the Canadian Occupational Performance Measure (21.2 %). Within the 30 reported interventions, 73.3% related directly to the person, 20% pertained to occupation (activities and participation), and 6.7% addressed environmental factors. The distinction and complementarity between the bottom-up and the top-down approaches to OT intervention were discussed. This review highlights OT specificity in adult CP management.

## 1. Introduction

The World Health Organization (WHO) recognizes chronic pain (CP) as a public health problem throughout the world. More than a symptom, CP is recognized as a disease in the International Classification of Diseases [1]. A recent systematic review estimated that the prevalence of CP worldwide was 30.3% for the global population [2]. The experience of pain interferes with different aspects of an individual's life [3], limiting their involvement in activities of daily living and their health-related quality of life [3]. Reid et al. [4] reported in a systematic review that interference with functioning and well-being are significantly associated with increasing pain severity (*p* ≤ 0.001). In fact, according to the International Association for the Study of Pain (IASP), one-third of people suffering from CP are unable or less able to maintain an independent and meaningful lifestyle due to their pain [5].

Being involved in meaningful life situations is a determinant of health and well-being and is itself an effective therapy [6]. According to these authors, occupation is everything people do to occupy themselves, including looking after themselves (self-care), enjoying life (leisure), and contributing to the social and economic fabric of their communities (productivity). Actually, the various definitions of occupation show some similarities with the concept of participation [7, 8] which refer more broadly to activities essential to survival as well as activities and roles necessary for well-being [7]. Furthermore, the International Classification of Functioning, Disability, and Health (ICF) [9] provides a common and shared understanding of functioning as well as a language that is multidisciplinary [10] and uses “disability” as an umbrella term which includes impairments (in body structures and functions), activity limitations, and participation restrictions [9]. Through their overarching focus on occupational performance and occupational engagement, occupational therapists make a unique contribution to pain prevention and management programs [11].

Enabling occupation is the ultimate goal of the OT clinical process. Occupational therapists provide a wide range of pain management interventions across the domains of physical, emotional, and spiritual occupational performance and have the knowledge, skills, and expertise to address CP management in all its complexity at individual clients and community and policy levels [11]. Occupational therapists' core skill, analyzing and adapting the physical, emotional, cognitive, and social demands of activities and occupations, allows them to identify and intervene on barriers to participate in specific life situations while considering environmental and personal factors [12–15].

Moreover, studies which describe the OT approach provide a deep understanding of the links between CP and occupation [16, 17] and suggest that engaging in occupation has the potential to mediate the pain experience and to alter biological, psychological, and social factors that are known to influence the pain experience [18, 19]. In addition to addressing the disruptions in occupational performance caused by CP [19, 20], occupational therapists are concerned with the occupational identity [12, 21] of their client. The Model of Human Occupation [22] states that “occupational identity” constitutes the values, beliefs, roles, and interests which drive a person to perform daily activities [12]. Indeed, occupational therapists recognize that CP affects all areas of a client's life and their competency and identity, and they are trained to use a holistic approach to address the occupational needs of clients living with this problem [12, 13, 21].

In spite of OTs theoretical foundations, many occupational therapists face challenges in defining and deploying their role in CP management. There appears to be a lack of a clear understanding by health professionals regarding the contribution of OT services with CP clients [18, 20]. Of further concern is the findings of Robinson et al. [19] who noticed and warned that occupational therapists tend to use inappropriate evidence in working with people with CP and rely too much on evidence developed in other disciplines such as psychology. For instance, they report that occupational therapists have added mindfulness-based approaches to their practice without critically addressing the contribution of this approach in enabling occupational performance and ultimately participation in life situations. Skjutar et al. [20] conclude that two actions are necessary to ensure the growth and development of OT services for clients with CP: (i) researchers must develop evidence of effectiveness on occupation-based outcomes and (ii) clinicians need to develop expertise in evidence-based practice.

More recently, Hesselstrand et al. [23] published a systematic review that assessed the quality of studies describing and evaluating the effects of OT intervention in adults experiencing CP. The main clinical recommendations arising from this review were that (i) OT interventions should start from the identified needs of the client with CP, (ii) no support exists for the effectiveness of electromyographic biofeedback training as a supplement, and (iii) efficacy of instructions on body mechanics was significant during work-hardening treatment. These recommendations can be misleading to health professionals as they are not specific to OT outcomes in CP which should endorse occupational engagement and participation.

To our knowledge, besides Hesselstrand et al. [23], no recent comprehensive review of OT practice in CP management is published. The purpose of the present review was to provide an overview of the current state of knowledge about the OT roles, models, assessments, and interventions methods used with adults living with CP.

## 2. Methods

Research in the field of rehabilitation tends to involve diverse methodological approaches and relies on both quantitative and qualitative data [24]. This heterogeneity and the widely dispersed publication of rehabilitation evidences pose substantial challenges for knowledge synthesis in rehabilitation [24]. A scoping review is a specific type of literature review involving rigorous and transparent methods for data collection, analysis, and interpretation [25]. This structured approach suits various research questions and facilitates gathering information from different sources, such as scientific literature and gray literature, as well as different study designs [26, 27]. Furthermore, scoping reviews aim to examine broad knowledge areas in order to identify gaps in the available scientific evidences; clarify key concepts; and report on relevant studies addressing and informing practice on a topic area [28]. Considering the need for a better understanding of the state of knowledge about OT for CP management in adults, a scoping review in the literature published between 2006 and 2017 was conducted according to the Arksey and O'Malley's framework [27] enhanced by the work of Levac et al. [26].

According to these authors, stage 1 involves identifying the research question. For this study, the main question was, “What is the current state of knowledge about OT for CP management in adults?” Three underlying questions based on the scientific literature contributed to documenting the OT process: (i) “What is the role of an occupational therapist for CP management in adults?” (ii) “Which assessment methods are available for use by the occupational therapist in CP management in adults?” (iii) “Which interventions support the OT intervention in CP management in adults?” The Canadian Practice Process Framework (CPPF) [6] formed the conceptual foundation for reporting the results.

Stage 2 involves identifying relevant studies. The scientific literature was searched through the following databases: Academic Search complete, CINAHL, Evidence-based medicine, Psychology and behavioral science collection, PsycINFO, PubMed, Scopus (MEDLINE and Embase), and OTseeker. The gray literature was searched through ProQuest for thesis and dissertations, and the Library University website was searched for books. Searching strategies were developed and adapted to the various databases as required in collaboration with an experienced librarian. The details of the search strategy used are given in Table 1.

Stages 3 and 4 involve an iterative team approach to selecting studies and charting data. To be included, articles had to meet all the following criteria: (i) document OT roles/assessments/interventions; (ii) focus on CP according to the WHO International Classification of Diseases (chronic primary pain, chronic posttraumatic and postsurgical pain, chronic neuropathic pain, chronic headache and orofacial pain, chronic visceral pain, and chronic musculoskeletal pain) [1]; (iii) address an adult population (18–65 years old); (iv) be published between 2006 and 2017; and (v) be written in English or French. Sources were excluded if they addressed cancer pain, hand therapy, and chronic diseases, and discussed only interdisciplinary or multidisciplinary approaches in CP management, without any specification about OT practice.

**Figure 1:**
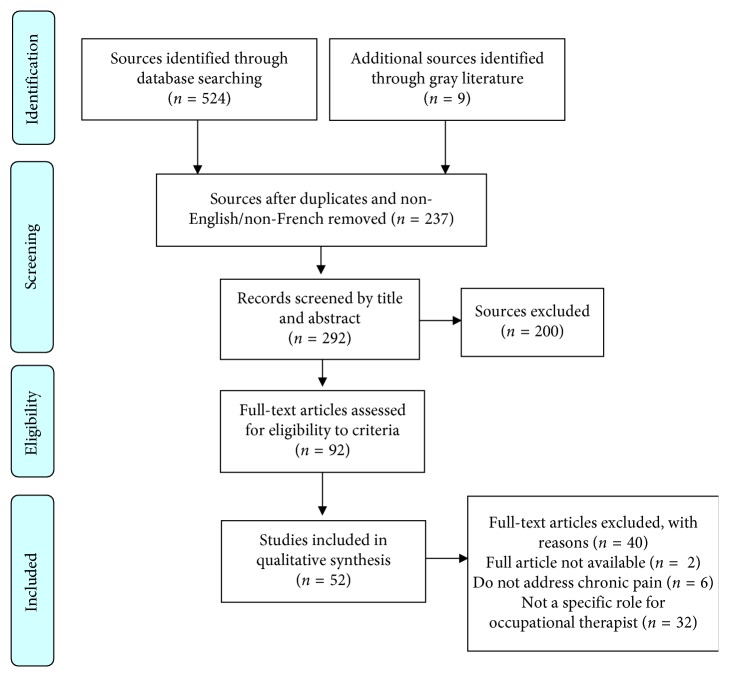
Flowchart of sources screened and included in the scoping review.

A total of 524 sources were identified through searching databases (53 from Academic Search complete, 60 from CINAHL, 12 from Evidence-based medicine, 9 from Psychology and behavioral science collection, 47 from PsycINFO, 94 from PubMed, and 243 from Scopus (MEDLINE and Embase, and 6 from OTseeker), and also 9 sources from potentially relevant gray literature were identified. Independently, two members of the research team (first and second authors) read each title/abstract and judged whether they were relevant to the research question and inclusion criteria or not. When a disagreement arose between the two reviewers, the third member of the research team (last author) offered additional consultation until a decision was reached. If the relevance of a study was still unclear, then the full article was read. Thus, 92 of the 533 sources were retained for full-text review. All 92 sources were reviewed by one member of the research team (second author) and independently validated by one researcher (first author). Hence, the three authors agreed on removing 40 sources considered irrelevant for the purpose of the study. Consequently, 52 sources were retained for data extraction. Two reviewers (second and last authors) extracted relevant information from these 52 sources, which were subsequently validated by the first author (first author). A flowchart of the source selection process for each stage of the review is presented in Figure 1.

The review team developed a data extraction process by analyzing three studies to ensure the presence of all relevant information. Charting the results was an iterative process whereby the charting table was continually updated [28]. This process leads to the addition of a category related to the models/conceptual frameworks used by the author(s). The final data chart included (i) author(s); (ii) year of publication; (iii) country of origin; (iv) type of publication; (v) aim/purpose; (vi) study population and sample size (if applicable); (vii) role of the occupational therapist; (viii) models/conceptual frameworks; (ix) OT assessment methods; and (x) OT interventions methods.

Stage 5 involves collating, summarizing, and reporting results. In the present case, qualitative thematic content analysis [29] was performed to describe OT practice for CP management in adults regarding roles, models/conceptual frameworks, assessment, and interventions methods. Frequency analysis was used to identify the number of sources and the characteristics related to the categories.

## 3. Results

A total of 52 sources were included in this scoping review: 19 reviews, 13 empirical studies, 7 qualitative studies, 6 documents from the gray literature, 3 descriptive studies, 3 author opinions, and 1 mixed-method study. These sources originated in the USA (*n*=21; 40.4%), Canada (*n*=8; 15.4%), Sweden (*n*=6; 11.5%), Spain (*n*=3; 7.8%), Australia, France, Ireland, Germany, Netherlands (*n*=2 for each of them; 3.8%), Belgium, Denmark, Swiss, and United Kingdom (*n*=1 for each of them; 1.9%).  Table 2 presents the synthesized results.

### 3.1. Occupational Therapy Roles

Occupational therapists in CP management suit different roles as shown in Table 3. They may be classified according to the ICF.

#### 3.1.1. Activities and Participation

The most frequently reported OT role in CP management (*n*=40/52; 76.9%) was to improve activities and participation and was distributed in the following way: enabling occupational engagement (*n*=20; 38.5%) and occupational performance (*n*=15; 28.8%), providing vocational rehabilitation (*n*=11; 21.2%), promoting participation (*n*=5; 9.6%), promoting functional independence, mobility, and autonomy (*n*=5; 9.6%), addressing occupational balance (*n*=4; 7.7%), restoring occupational identity (*n*=2; 3.8%), and limiting occupational injustice (*n*=1; 1.9%).

#### 3.1.2. Body Functions and Structures

Improving and restoring function (*n*=5; 9.6%), as well as improving body mechanics and activity tolerance (*n*=2; 3.8%), were mentioned as part of the OT role in CP management.

#### 3.1.3. Environmental Factors

OT involvement in modifying the nonhuman environment and adopting an ergonomic approach (*n*=17; 32.7%) and in enhancing social support (*n*=8; 15.4%) for adults with CP was mentioned by some authors.

#### 3.1.4. Personal Factors

Most of the sources pertained to general CP in adults (*n*=28; 53.8%), followed by chronic low back pain (*n*=10; 19.2%), fibromyalgia (*n*=6; 11.5%), complex regional pain syndrome (*n*=5; 9.6%), headaches (*n*=2; 3.8%), and chronic shoulder pain (*n*=1; 1.9%).

### 3.2. Occupational Therapy Models and Conceptual Frameworks

The models and conceptual frameworks reported are summarized in Table 4. In order to specifically address occupational issues linked to CP, the authors directed their attention towards disciplinary OT models of practice and practice frameworks. The ones that were mentioned the most where the Canadian Model of Occupational Performance (CMOP) (*n*=5; 9.6%) and the Canadian Model of Occupational Performance and Engagement (CMOP-E) (*n*=1; 1.9%). The CMOP specifies three core constructs of interest for the profession of OT (persons, environments, and occupations) and portrays occupational performance as the result of their dynamic interaction [11, 76]. To extend its occupation-based perspective, engagement was added as a conceptual advancement on the original model [77]. Other models of practice are reported such as the Occupational Therapy Practice Framework: Domain and Process (*n*=7; 13.5%). This practice framework provides a systematic method of combining a variety of theoretical conceptual practice models to address a client's issues more comprehensively and guides occupational therapists towards combining multiple theoretical models to address client occupational performance issues and in defending their clinical decisions effectively [78].

### 3.3. Occupational Therapy Assessments

Table 5 summarizes the main domains of assessment methods of occupational therapists in CP management. The most reported measurement tool was the Canadian Occupational Performance Measure (COPM) (*n*=11; 21.2%). Other evidence-based outcome assessments included the Occupational Performance History Interview (OPHI-II) (*n*=3; 5.8%) and the Assessment of Pain and Occupational Performance (POP) (*n*=2; 3.8%). The following assessment methods were mentioned in only one source: the Functional Independence Measure (FIM) [60]; the Milliken Activities of Daily Living Scale (MAS) [47]; the Assessment of Motor and Process Skills (AMPS) [75]; the Assessment of Life Habits Questionnaire (LIFE-H) [68]; the Impact on Participation and Autonomy (IPA) [58]; and the Pain and Functional Performance Assessment (PFPA) [38].

### 3.4. Occupational Therapy Interventions

OT interventions in CP management were classified according to the intervention taxonomy developed by McColl and Law [79] which categorises eight types of interventions used by occupational therapists to enable occupation as shown in Table 6.

#### 3.4.1. Focus on Person

The most frequently reported interventions are related to body mechanics as well as posture and positioning (*n*=14; 26.9%). Energy conservation and joint-sparing techniques (*n*=9; 17.3%), relaxation training and stress management (*n*=8; 15.4%), and exercises or fitness programs (*n*=7; 13.5%) were also mentioned as interventions used in CP management. Moreover, six sources (*n*=6; 11.5%) included mindfulness or cognitive behavioral therapy and behavioral approaches. Five sources (*n*=5; 9.6%) involved one of the following categories: coping strategies; coordination, dexterity, and strengthening tasks; desensitization techniques; and sensory reeducation. Three sources (*n*=3; 5.8%) presented one of the following categories: active movements and mobilization techniques; biofeedback; functional splinting; oedema modalities; and proprioceptive neuromuscular facilitation. Two sources (*n*=2; 3.8%) suggested one of the following categories: thermal modalities; graded motor imagery; mirror visual feedback or stress loading. Finally, one source (*n*=1; 1.9%) mentioned breathing techniques, electrical stimulation, massage and acupressure, and mental imagery and visualization.

#### 3.4.2. Focus on Environment

The most frequently reported OT interventions addressing the environment related to ergonomics (*n*=16; 30.8%) and 11 sources (*n*=11; 21.2%) are mentioned environmental modification.

#### 3.4.3. Focus on Occupation

Across all intervention categories, pacing and graded activity (*n*=19; 36.5%) and activity (task) adaptation (*n*=17; 32.7%) were the most frequently reported OT interventions. Vocational intervention (*n*=14; 26.9%), reeducation, and sleep hygiene (*n*=3; 5.8%) were also mentioned as occupation-focused OT interventions. Finally, two sources (*n*=2; 3.8%) suggested one of the following categories: graded in vivo exposure, yoga, and Tai Chi.

## 4. Discussion

In this scoping review, current knowledge about OT in CP management is described after collating and analyzing scientific literature and gray literature about the OT roles, models, assessments, and interventions methods. The results offer insight about OT practice specific to CP management.

### 4.1. Role

The analysis suggests that occupational therapists mainly aim to improve activities and participation with adults living with CP. This aim is particularly consistent with the top-down approach which adopts a more global perspective of the client's participation in his or her living contexts while considering what is important and relevant to him or her [81]. This aim is aligned with the “participation” level of the ICF [82] and intimately connects to the evidence-based relationship between occupation and health as well as fundamental assumptions in OT regarding how occupations influence people's health and vice-versa [10, 83]. By primarily identifying participation restrictions and understanding their causes, the top-down approach leads to a participation-oriented intervention which can provide CP clients with the support needed for involvement in their daily activities despite pain and physical impairments, such as a lack of strength or insufficient range of motion.

Whereas a biomedical model of health encourages a technique-oriented practice, an occupation-based practice emphasizes the use of occupation to address the occupational consequences of CP. Our findings show that improving and restoring function as well as improving body mechanics and activity tolerance is part of the aims of occupational therapists in CP management (see Table 3; body functions and structures). An understanding of whether occupational therapists' specificity emerges here while the bottom-up approach to OT interventions is put forward is particularly pressing. Indeed, in 2011, Robinson et al. [67] warned the scientific community that the influence of the biomedical model of health on OT limited the integration of occupation-based practice for people with CP, the latter being the central value of OT. More recently, Burley et al. [84] also witnessed two competing perspectives, biomechanical and occupational, that are present in OT services in the hand therapy literature. They noticed that whilst there has been some integration of an occupational perspective, a bottom-up approach, an inconsistent use of terminology to describe what could be framed as occupations, and a lack of an occupation-based performance perspective persist throughout the OT clinical process. Although this population is not included in this study, these findings demonstrate the continuing tensions between the biomechanical approach and an occupational perspective that can be experienced in the OT profession. Therefore, the best practice requires that occupational therapists thoughtfully elaborate their clinical process without neglecting the purpose and focus of OT, enabling occupation.

### 4.2. Models

The interactions between CP and occupation are complex and go well beyond work since personal care, sleep, and family life may also be affected. This is the rational supporting the notion that the complexity of the interactions between all domains of occupation be considered when occupational therapists intervene [65]. Complementary to the biopsychosocial (model or approach), occupation-focused models such as the CMOP/CMOP-E support a holistic understanding and guide the intervention towards the occupational challenges that adults living with CP face [85]. Moreover, by identifying occupational engagement as an important aspect of human occupation, the CMOP/CMOP-E aligns with the current developments and improvements in knowledge related to occupation-based, client-centered and evidence-based OT practice [77]. Occupational engagement considers meaning, interest, motivation, and/or perceived self-efficacy as criteria necessary for a client-centered practice [6] all of which are of particular interest in the context of CP. The way in which individuals with CP engage in their daily activities has been shown to impact on daily functioning. Indeed, a growing body of OT research has begun to explore the complexity of the occupational experiences of people living with CP [65, 86] as well as its impact on quality of life [3]. In fact, the relationship between the levels of engagement in occupations and pain experience has been explored [16, 17]. It has been found that both avoidance of activities and overactivity are associated with more pain, higher levels of physical disability, and poorer psychological functioning [17].

In sum, evidence exists to support the need for OT as an intervention which explicitly endorses an occupation-based perspective. By considering the occupational aspect of living with CP, occupational therapists strengthen and differentiate their role thereby affirming OT specificity within a multidisciplinary team [77].

### 4.3. Assessment Methods

Historically, bottom-up assessments are frequently used in OT practice and fit within the biomedical model [87]. The approach of using standardized tests and emphasizing physical impairments and disabilities and subsequently inferring their influence on participation restriction may be conducive to communication and may enable a transdisciplinary approach to intervention. However, it is flawed reasoning. Indeed, widespread clinical belief that improvement in body structures and functions reduces activity limitations and participation restrictions has not been convincingly demonstrated in the literature [88]. This scoping review has exposed the challenges of distinguishing the concepts of “activities” and “participation” described in the ICF model. Unfortunately, these concepts are often combined and confounded in contrast with those of body structures and function [89]. For example, questionnaires are typically used to measure the perception of physical functioning in the presence of pain [90] and not the true level of participation. It is important for researchers to keep in mind that participation, which is the concept most closely linked to the occupation-based perspective of OT, differs from activity. Participation refers to involvement in real life situations instead of the execution of a task or action [9], measuring participation can be a challenge but validated tools do exist such as the COPM and the OPHI-II [91] as presented in this review. Occupational therapists should therefore be leaders in implementing these tools in their practice with the adult CP population.

Occupational therapists can evaluate how pain influences the way an individual performs and engages in his daily occupations [13]. However, another angle that should be assessed is how performance and engagement in daily occupations affect health and well-being in the presence of CP. Our findings show that the most frequently reported assessment method was the COPM. This evidence-based outcome measure is designed to detect change in the client's self-perception of performance and satisfaction in self-care, productivity, and leisure occupations over time [92] and has recently been used to the measure the efficacy of an interdisciplinary CP rehabilitation program [51]. By combining different assessment methods such as activity analysis, occupational interviews, observations, and standardized functional tests, occupational therapists are skilled to observe and understand the interaction between multiple personal, environmental, and occupational factors that explain the gap between what a person living with CP wants and needs to do and their level of participation [35, 93].

### 4.4. Interventions Methods

On top of having tools and expertise to quantify and objectively measure occupational performance and engagement (functional performance), occupational therapists also are experts at restoring function through purposeful and meaningful occupations [94]. This intervention was supported by this scoping review which exposed that of the 30 different OT interventions in CP management, seventy-three percent of them related directly to the person, 20% pertained to activities and participation, and 7% addressed environmental factors. Interestingly, the two most frequently discussed interventions refer to an occupation-based intervention: pacing and graded activity (36.5%) and activity (task) adaptation (32.7%).

People with CP show lifestyle and work-related issues which suggest a need for interventions which consider the complex relationships between the person, environment, and occupations [95]. However, in the last few years, little research about the efficacy of OT interventions in CP management has been conducted despite explicit recommendations [11, 19]. Considering evidence that engagement in occupation is essential for health and well-being, new studies in the field of OT will enforce the unique contribution of occupational therapists to the management of CP. Occupation-based interventions do exist; for instance, Simon and Collins [71] recently suggested that a specific OT intervention optimizes participation in integrated occupations and could contribute to CP management. Also of interest, for CP adults, could be training in Lifestyle Redesign® which is a manualized OT intervention grounded in occupational science research. This training technique focuses on facilitating client development of healthy self-care routines and habits to prevent and manage chronic conditions. According to these authors, this manualized intervention has demonstrated significant changes in occupational performance and satisfaction scores, physical and social functioning, role limitations due to physical and emotional problems, energy and fatigue, general health, and pain self-efficacy in adult with CP. Other studies of this kind are essential to expose to the health professional community research known to scientifically support OT practice interventions reflecting OT specificity in CP management.

### 4.5. Future Implications

According to Cronin and Mandich [88], occupational therapists need to be fluent in both the bottom-up and the top-down approaches and understand their strengths and weaknesses within different clinical situations. The bottom-up and the top-down approach to evaluation and to intervention are complementary and prioritized according to the needs of the client and clinical setting and considering the client's stage of rehabilitation and readiness to address specific types of problems [96]). However, occupational therapists should take warrant that the historic tendency to relying too much on the bottom-up approach might in some cases forego or at least reduce the OT impact on participation. To promote a better understanding of the centrality of occupation, when choosing either a top-down or bottom-up approach, occupational therapists should seek to explicitly link their intervention to long-term outcomes linked to occupational performance and engagement or participation. This is a crucial challenge for occupational therapists especially if a bottom-up approach is privileged. By relentlessly reminding health professionals that OT focus is on occupation as a mean and an end, the profession will continue to strongly position itself as a valuable and distinctive contribution to adult CP management.

### 4.6. Study Strengths and Limitations

In this study, we meticulously followed the first five steps recommended to conduct a scoping review and explored a variety of sources to ensure that the most relevant documents were included. Covalidation of data charting and analyses also ensured that interpretation of the findings was valid. However, the inclusion of stage six, although optional, would have made it possible to provide opportunities for consumer and stakeholder involvement to suggest additional references and provide insights beyond those in the literature [26]. This has partly been addressed by a covalidation process that has been put in place within the research team and two collaborators. Also, despite an extensive search, some relevant documents may have been missed. Moreover, the quality of the publications included in this review was not critically appraised because this criterion is not one of the objectives of scoping reviews. Nonetheless, we are confident that the current study provides a clear picture of current knowledge and is a good starting point to orient future research and helps understanding and promoting OT in CP management knowing that the referral to an occupational therapist depends on other health care professionals' knowledge and understanding about OT [95].

## 5. Conclusion

In this scoping review, four themes linked to the current state of knowledge about OT in CP management emerged: (i) as an expert in enabling occupation, occupational therapists are particularly interested in how individuals living with CP can perform and engage in their daily occupations, (ii) the use of disciplinary models ensures a better emphasis on occupation as an end and as a mean throughout the entire OT treatment process, (iii) the top-down approach which is more in line with OT specific values and skills is complementary to the bottom-up approach, and iv) further studies about occupation-based interventions and their effectiveness are required to better address occupational issues which are facing adults living with CP and enrich the entire OT practice process by bringing forward its specific contribution to the optimization of the participation of people facing occupational challenges related to living with CP on a daily basis.

## Figures and Tables

**Table 1:** Database Search strategy.

Database	Search strategy
Academic search complete, CINALH, evidence-based medicine, psychology and behavioral science collection, PsycINFO, and Scopus (MEDLINE and Embase)	(Occupational therapy: “Occupational therap∗”/) AND (chronic pain related terms: “Chronic pain∗” OR fibromyalgia∗ OR “back pain∗” OR “long term pain∗” OR migraine∗ OR “cervical pain∗” OR “neck pain∗” OR “complex regional pain syndrome” OR “chronic musculoskeletal∗ pain∗” OR “chronic headache∗” OR “chronic orofacial∗ pain∗” OR “chronic neuropathic∗ pain∗”) AND (practice related terms: Intervention∗ OR evaluat∗ OR assess∗ OR role∗ OR manag∗)

PubMed	(Occupational therapy: Occupational therapy OR occupational therapist OR occupational therapies OR occupational therapists) AND (chronic pain related terms: “Chronic pain” OR “chronic pains” OR fibromyalgia OR fibromyalgias OR “back pain” OR “back pains” OR migraine OR migraines OR “cervical pain” OR “cervical pains” OR “neck pain” OR “neck pains” OR “complex regional pain syndrome” OR “chronic musculoskeletal pain” OR “chronic musculoskeletal pains” OR “chronic headache” OR “chronic headaches” OR “chronic orofacial pain” OR “chronic orofacial pains” OR “chronic neuropathic pain” OR “chronic neuropathic pains”) AND (practice related terms: Intervention OR interventions OR evaluate OR evaluation OR evaluations OR assess OR assessment OR role OR roles OR manage OR management)

OTseeker	(Practice related terms: Assess∗ OR role∗ OR manag∗ OR evaluat∗ OR intervention∗ AND “chronic pain∗”)

**Table 2:** Summary of publications in the final analysis (*n*=52).

Author(s), year, country of origin	Aim/purpose	Sample size	Study population	Type of publication
Aegle and Satink [30] Netherlands	To explore how persons with chronic pain experienced their occupational performance.	*n*=8 subjects	CP	Qualitative study

Artner et al. [31] Germany	To introduce and evaluate the short-term outcome of a three-week intensive multidisciplinary outpatient program for patients with back pain and sciatica, measured according to decrease of functional impairment and pain.	*n*=160 subjects	Chronic LBP	Pilot study (retrospective analysis)

Ashby et al. [32] Australia	To examine the difficulties men with chronic LBP experienced in the maintenance of their leisure activities and to explore the connection between leisure and social networks and the potential barriers to resuming leisure occupations due to chronic LBP.	*n*=11 patients (men)	Chronic LBP	Ethnographic study

Bosy et al. [33] Canada	To describe the essential elements of an intensive 8-week interdisciplinary pain rehabilitation programme with a cognitive-behavioral emphasis and the results that can be expected in treating patients with chronic pain conditions.	*n*=338 patients	CP	Observational study

Caby et al. [34] France	To evaluate the efficiency of an intensive, dynamic, and multidisciplinary functional restoration program in patients with chronic LBP, during 6-month follow-up and 12-month follow-up.	*n*=144 patients	Chronic LBP	Retrospective, nonrandomized controlled study

Cammalleri [35] Canada	To specifically look at the relationships between religion and pain experiences.	*n*=2 women	CP	Gray literature/dissertation qualitative study

Demoulin et al. [36] Belgium	To evaluate the efficacy of a semi-intensive multidisciplinary outpatient program complying with the requirements of the Belgian National Institute for Health and Disability Insurance and intended for patients with chronic LBP.	*n*=262 patients	Chronic LBP	Pretest-posttest with a control group

Dobkin et al. [37] Canada	To identify factors associated with decreased disability and lower pain scores 6 months after a multimodal treatment program for FM and to determine whether adherence influenced outcomes.	*n*=46 women	FM	Quasiexperimental study

Fisher et al. [13] USA	To explore the lived occupational experiences of people who have chronic pain.	*n*=13 patients	CP	Qualitative phenomenological study

Fisher et al. [38] USA	To develop an occupation-based assessment that measured and described the occupational performance of individuals with CP.	*n*=25 patients +17 practicing occupational therapists	CP	Methodological study

Gallice et al. [39] Swiss	To present the practice of a multidisciplinary functional rehabilitation program for patients with chronic LBP.	—	Chronic LBP	Author opinion

Gatchel and Dougall [40] USA	This chapter reviews and discusses data demonstrating the close comorbidity between chronic musculoskeletal and mental health disorders.	—	CP	Gray literature book chapter 12

Lippe and Polatin [41] USA	This chapter describes the basic rationale behind an interdisciplinary approach, the interdisciplinary program framework, and the role that this approach plays when considering disability in the workplace.	—	CP	Gray literature book chapter 20

Chapman et al. [42] USA	This chapter discusses four important areas related to vocational assessment and training for patients with chronic musculoskeletal disorders.	—	CP	Gray literature book chapter 25

Gonzalez et al. [43] Spain	To evaluate the improvement of ADL and quality of life following a multidisciplinary intervention (health primary care and occupational therapy).	*n*=21 patients	FM	Pretest-posttest study

Harden et al. [44] USA	To provide treatment guidelines for CRPS.	—	CRPS	Literature review

Harden et al. [45] USA	To provide diagnostic and treatment guidelines for CRPS.	—	CRPS	Literature review

Hardison and Roll [46] USA	To describe how mindfulness is used in physical rehabilitation, identify implications for OT practice, and guide future research on clinical mindfulness interventions.	*n*=16 sources	CP	Scoping review

Hesselstrand et al. [23] Sweden	To assess the quality of studies describing and evaluating the effects of OT interventions on CP.	*n*=19 studies	CP	Systematic literature review

Hill [12] United Kingdom	To explain the role and value of OT within the pain management team.	—	CP	Author opinion

Jarrar [47] USA	To evaluate the effects of mirror therapy on upper extremity pain and function for individuals with CRPS.	*n*=1 (case study)	CRPS	Gray literature/thesis systematic review and case study

Kallhed and Mårtensson [48] Sweden	To explore how persons with CP reason about their use and choice of strategies to manage activities of everyday life.	*n*=8 persons	CP	Qualitative study (semistructured interviews)

Keponen and Kielhofner [49] USA	To examine how women experience occupations as they live with CP and more specifically to gain detailed knowledge regarding the meaning of important occupations in their life.	*n*=17 narratives	CP	Qualitative study (narrative study)

King [50] USA	To discuss the nonpharmacologic therapies for managing CP.	—	CP	Literature review

Kurklinsky et al. [51] USA	To examine the efficacy of interdisciplinary rehabilitation for improving function in people with CP.	*n*=150 patients	CP	Retrospective chart review

Linden et al. [52] Germany	To evaluate the effectiveness of cognitive behaviour group therapy in respect to pain tolerance and disability apart from the effects on somatization in general and additional to the effects of a multimodal inpatient orthopedic rehabilitation program.	*n*=103 patients (53 patients with chronic LBP and 50 controls)	Chronic LBP	Randomized controlled trial

Mathews [53] USA	To discuss the principles of CP rehabilitation and some of these modalities in greater detail.	—	CP	Literature review

McCormack and Gupta [54] USA	To discuss and illustrate the use of complementary approaches to pain management by occupational therapists.	—	CP	Literature review

McLean [55] Canada	To report tools those occupational therapists or other clinicians may use to provide support to migraineurs (and their employer) in the workplace.	—	Headache	Literature review

McLean et al. [56] Canada	To review the pacing literature; describe the use of pacing in a specialty headache clinic; and provide client feedback regarding the effectiveness of pacing in headache self-management.	*n*=20 patients	Headache	Literature review

Miles [57] USA	To propose a multidisciplinary pain treatment program that aims to reduce the pain and improve the functioning of the patient, as well as to improve the communication between specialists to facilitate patient progress.	—	CP	Gray literature/dissertation mixed-methods study

Nieuwenhuizen et al. [58] Netherlands	To examine the construct validity and construct responsiveness of the Dutch version of the COPM performance scale in a population with CP.	*n*=87 patients	CP	Methodological study

Paquette [59] USA	To apply the OT framework along with an evidence-based approach and an occupation-based intervention with a population of workers with chronic LBP to help them return to work and maintain their work status.	—	Chronic LBP	Literature review

Pérez de Heredia-Torres et al. [60] Spain	To evaluate the differences in cognitive skills between women with FM and healthy women, and the correlations between functional independence and cognitive limitations.	*n*=40 patients (20 controls, 20 patients with FM)	FM	Cross-sectional case control study

Perneros and Tropp [61] Sweden	To present the development of The Assessment of Pain and Occupational Performance and to evaluate validity and reliability.	*n*=220 patients (142 chronic LBP, 97 specific LBP, 45 nonspecific back pain)	Chronic LBP, specific LBP and nonspecific back pain	Descriptive longitudinal study

Perneros et al. [62] Sweden	To evaluate occupational performance and pain intensity in daily occupations for patients with chronic LBP.	*n*=97 patients	Chronic LBP	Descriptive longitudinal study

Persson et al. [63] Sweden	To describe everyday occupational problems among patients with musculoskeletal pain enrolled in a pain rehabilitation programme, and to compare subgroups based on participant characteristics.	*n*=152 participants	CP	Mixed-methods study

Poole and Siegel [64] USA	To summarize evidence focused on effectiveness of OT-related interventions for adults with FM.	*n*=42 sources	FM	Systematic review

Prefontaine and Rochette [65] Canada	To review the relationship between CP and engagement in instrumental ADL, sleep, and family life.	*n*=13 sources	CP	Literature review

Ravenek et al. [66] Canada	To update the evidence for the multidisciplinary treatment of chronic LBP to improve employment outcomes and to assess what knowledge supports OT as contributing to a multidisciplinary approach in the treatment of chronic LBP.	*n*=12 sources	Chronic LBP	Systematic review

Robinson et al. [67] Ireland	To critically analyse OT services for people with CP and identify significant factors influencing the future development of OT services for people with CP.	—	CP	Literature review

Robinson et al. [19] Ireland	To discuss contemporary OT for people with CP with reference to a broad range of literature from many disciplines, and to examine the success of OT services in meeting the occupational needs of people with CP.	—	CP	Author opinion

Rome [68] France	To assess the value of combining OT with physical therapy for the rehabilitation of CRPS and to measure its effectiveness on ADL.	*n*=60 patients	CRPS type 1	Comparative cases study

Salgueiro et al. [69] Spain	To evaluate the ability of artificial neural networks to predict, on the basis of clinical variables, the response of persons with FM syndrome to a standard, 4-week interdisciplinary pain program.	*n*=71 patients	FM	Retrospective longitudinal study

Silvestri [21] USA	To examine the implications of chronic shoulder pain on quality of life and occupational engagement in spinal cord injury.	—	Chronic shoulder pain	Literature review

Simon and Collins [70] USA	To determine the efficacy of a lifestyle Redesign® intervention for people living with CP on quality of life, function, self-efficacy, and pain levels.	*n*=45 patients	CP-chronic LBP, myalgia (FM), and CRPS	Retrospective study

Skjutar et al. [20] Sweden	To explore occupational therapists' perceptions of indicators for OT interventions among patients with CP.	*n*=25 occupational therapists	CP	Qualitative study (focus group)

Stanos [71] USA	To examine components of interdisciplinary pain rehabilitation programs, to discuss desirable features of successful programs and teams, and to review four established outpatient pain programs in the United States.	*n*=4 (program)	CP	Focused review

Stewart et al. [72] USA	To offer perspectives from life care planners, an occupational therapist, rehabilitation counsellors, and a pain management specialist on CP treatment.	—	CP	Literature review

Tran et al. [73] Canada	To summarize the evidence derived from randomized controlled trials pertaining to the treatment of CRPS.	*n*=41 sources	CRPS	Narrative review

van Huet et al. [74] Australia	To explore factors that contribute to clients' CP management from an OT perspective.	*n*=9 occupational therapists	CP	Qualitative study (narrative inquiry)

Von Bülow et al. [75] Denmark	To identify frequently reported ADL skill deficits of significance in subgroups of women with FM who have decreased ADL motor ability in combination with decreased or competent ADL process ability.	*n*=188 patients	FM	Cross-sectional study

Note. ADL = activities of daily living; COPM = Canadian occupational performance measure; CP = chronic pain; CRPS = complex regional pain syndrome; FM = fibromyalgia; LBP = low back pain; OT = occupational therapy.

**Table 3:** Occupational therapy roles in chronic pain management.

Roles	Authors
*Activities and Participation*	
Enabling occupational engagement	Ashby et al. [32]; Cammalleri [35]; Fisher et al. [13]; Fisher [38]; Kallhed and Mårtensson [48]; Keponen and Kielhofner [49]; King [50]; Linden et al. [52]; Mathews [53]; McCormarck, and Gupta [54]; McLean [55]; Miles [57]; Paquette [59]; Perneros et al. [62]; Prefontaine and Rochette [65]; Robinson et al. [19]; Robinson et al. [67]; Silvestri [21]; Skjutar et al. [20]; Stewart et al. [72]
Addressing occupational performance	Fisher et al. [13]; Gonzalez et al. [43]; Hesselstrand et al. [23]; Hill [12]; Jarrar [47]; Pérez de Heredia-Torres [60]; Perneros and Tropp [61]; Perneros et al. [62]; Persson et al. [63]; Poole and Siegel [64]; Prefontaine and Rochette [65]; Robinson et al. [19]; Robinson et al. [67]; Skjutar et al. [20]; Von Bülow et al. [75]
Providing vocation rehabilitation	Bosy et al. [33]; Demoulin et al. [36]; Gatchel and Dougall [40]; Chapman et al. [42]; Hesselstrand et al. [23]; Linden et al. [52]; Paquette [59]; Perneros et al. [62]; Prefontaine and Rochette [65]; Ravenek et al. [66]; Robinson et al. [19]
Promoting participation	Hill [12]; Kallhed and Mårtensson [48]; Kurklinsky et al. [51]; Perneros et al. [62]; Robinson et al. [19]
Promoting functional independence, mobility, and autonomy	Lippe and Polatin [41]; Kurklinsky et al. [51]; Miles [57]; Rome [68]; van Huet et al. [74]
Addressing occupational balance	Skjutar et al. [20]; Simon and Collins [70]; Kurklinsky et al. [51]; Kallhed and Mårtensson [48]
Restoring occupational identity	Hill [12]; Silvestri [21]
Limiting occupational injustice	Silvestri [21]

*Body functions and structures*	
Improving/Restoring function	Harden et al. [44]; Harden et al. [45]; Jarrar [47]; King [50]; Mathews [53]
Improving body mechanics and activity tolerance	Miles [57]; Stanos [71]

*Environmental factors*	
Modifying the nonhuman environment and ergonomic approach	Artner et al. [31]; Bosy et al. [33]; Caby et al. [34]; Chapman et al. [42]; Demoulin et al. [36]; Gallice et al. [39]; Hesseltrand et al. [23]; Hill [12]; Kallhed and Mårtensson [48]; Kurklinsky et al. [51]; Mathews [53]; Miles [57]; Ravenek et al. [66]; Robinson et al. [19]; Salgueiro et al. [69]; Stanos [71]; Stewart et al. [72]
Enhancing social support	Ashby et al. [32]; Gonzalez et al. [43]; Hill [12]; Paquette [59]; Perneros and Tropp [61]; Perneros et al. [62]; Simon and Collins [70]; Ravenek et al. [66]

**Table 4:** Occupational therapy models of practice and practice framework in chronic pain management.

Occupation-based model/framework	Authors
*Models of Practice*	
Canadian Model of Occupational Performance (CMOP)	Cammalleri [35]; Jarrar [47]; Nieuwenhuizen et al. [58]; Perneros and Tropp [61]; Persson et al. [63]
Canadian Model of Occupational Performance and Engagement (CMOP-E)	Prefontaine and Rochette [65]
Model of Human Occupation (MOHO)	Keponen and Kielhofner [49]
Ecology of Human Performance (EHP) Model	Silvestri [21]
Occupational Performance Model (OPM)	McLean et al. [56]
Kawa Model	Cammalleri [35]
Value and Meaning in Occupations (ValMO)	Kallhed and Mårtensson [48]
Dynamic Occupation in Time (DOiT) Model	Aegle and Satink [30]

*Practice Framework*	
Occupational Therapy Practice Framework: Domain and Process	Fisher et al. [38]; Hardison and Roll [46]; Jarrar [47]; McCormack and Gupta [54]; Paquette [59]; Poole and Siegel [64]; Simon and Collins [70]

**Table 5:** Occupational therapy assessments in chronic pain management.

Occupational therapy-used assessments	Authors
*Evidence-based outcome measure*	
Canadian Occupational Performance Measure (COPM)	Fisher et al. [38]; Hesselstrand et al. [23]; Jarrar [47]; Kurklinsky et al. [51]; Nieuwenhuizen et al. [58]; Perneros et al. [62]; Persson et al. [63]; Prefontaine and Rochette [65]; Simon and Collins [70]; Stanos [71]; van Huet et al. [74]
Occupational Performance History Interview (OPHI-II)	Fisher et al. [38]; Keponen and Kielhofner [49]; Prefontaine and Rochette [65]
Assessment of Pain And Occupational Performance (POP)	Perneros and Tropp [61]; Perneros et al. [62]
Functional Independence Measure (FIM)	Pérez de Heredia-Torres et al. [60]
Milliken Activities of Daily Living Scale (MAS)	Jarrar [47]
Assessment of Motor and Process Skills (AMPS)	Von Bülow et al. [75]
Assessment of Life Habits Questionnaire (LIFE-H)	Rome [68]
Impact on Participation and Autonomy (IPA)	Nieuwenhuizen et al. [58]
Pain and Functional Performance Assessment (PFPA)	Fisher et al. [38]

*Assessment Domain*	
Work (vocational) assessment or job-site analysis or ergonomic Work Assessment	Bosy et al. [33]; Chapman et al. [42]; Gallice et al. [39]; Harden et al. [44]; Harden et al. [45]; Hesselstrand et al. [23]; Hill [12]; McLean [55]; Ravenek et al. [66]; Stanos [71]; van Huet et al. [74]
Analysis of occupational performance, occupation, role, activity and/or participation in ADL	Ashby et al. [32]; Fisher et al. [38]; Hesselstrand et al. [23]; Hill [12]; Jarrar [47]; McCormack and Gupta [54]; Mathews [53]; Perneros and Tropp [61]; Perneros et al. [62]
Functional capacity (abilities and limitations) evaluation	Gallice et al. [39]; Harden et al. [44]; Harden et al. [45]; Hesselstrand et al. [23]; Paquette [59]; Stanos [71]
Occupational profile/occupational history	Mathews [53]; McCormack and Gupta [54]
Work capacity evaluation/transferable skills analysis	Harden et al. [44]; Harden et al. [45]
Home assessment	Hill [12]; van Huet et al. [74]

**Table 6:** Occupational therapy interventions in chronic pain management.

Treatment methods	Authors
*Focus on PERSON* (training, skill development, and education)	
Body mechanics/postures and positioning	Bosy et al. [33]; Demoulin et al. [36]; Dobkin et al. 2010 [37]; Cammalleri [35]; Chapman et al. [42]; Kurklinsky et al. [51]; Mathews [53]; Nieuwenhuizen et al. [58]; Robinson et al. [19]; Simon and Collins [70]; Stanos [71]; Stewart et al. [72]; Tran et al. [73]; van Huet et al. [74]
Energy conservation/joint-sparing techniques	Bosy et al. [33]; Cammalleri [35]; Hesselstrand et al. [23]; Hill [12]; McLean [55]; Robinson et al. [19]; Simon and Collins [70]; Skjutar et al. [20]; van Huet et al. [74]
Relaxation training/stress management	Fisher et al. [13]; Hill [12]; McLean [55]; Poole and Siegel [64]; Robinson et al. [19]; Robinson et al. [67]; Simon and Collins [70]; van Huet et al. [74]
Exercises/fitness program	Cammalleri [35]; Kallhed and Mårtensson [48]; Paquette [59]; Poole and Siegel [64]; Ravenek et al. [66]; Robinson et al. [19]; Simon and Collins [70]
Mindfulness	Hardison and Roll [46]; Hesselstrand et al. [23]; Kallhed and Mårtensson [48]; McCormack and Gupta [54]; Poole and Siegel [64]; Robinson et al. [19]
Cognitive behavioral therapy/behavioral approaches	Miles [57]; Poole and Siegel [64]; Robinson et al. [19]; Robinson et al. [67]; Simon and Collins [70]; van Huet et al. [74]
Coping strategies	Fisher et al. [13]; Gatchel and Dougall [40]; Hill [12]; Skjutar et al. [20]; Stewart et al. [72]
Coordination/dexterity, strengthening tasks	Harden et al. [44]; Harden et al. [45]; Jarrar [47]; Mathews [53]; Rome [68]
Desensitization techniques/sensory reeducation	Harden et al. [44]; Jarrar [47]; Mathews [53]; Rome [68]; Tran et al. [73]
Active movements/mobilization techniques	Harden et al. [44]; Harden et al. [45]; Robinson et al. [19]
Biofeedback	Kurklinsky et al. [51]; Ravenek et al. [66]; Robinson et al. [19]
Functional splinting	Harden et al. [44]; Robinson et al. [19]; Robinson et al. [67]
Oedema modalities	Harden et al. [44]; Harden et al. [45]; Tran et al. [73]
Proprioceptive neuromuscular facilitation/reeducation	Harden et al. [44]; Harden et al. [45]; Stewart et al. [72]
Thermal modalities	Cammalleri [35]; Robinson et al. [19]
Graded motor imagery	Harden et al. [45]; Tran et al. [73]
Mirror visual feedback	Harden et al. [45]; Jarrar [47]
Stress loading	Harden et al. [44]; Harden et al. [45]
Breathing techniques	McCormack and Gupta [54]
Electrical stimulation	Robinson et al. [19]
Massage/acupressure	McCormack and Gupta [54]
Mental imagery/visualization	McCormack and Gupta [54]

*Focus on ENVIRONMENT* (environmental modification, support provision, and support enhancement)	
Ergonomics (home, work, and equipment)	Artner et al. [31]; Bosy et al. [33]; Caby et al. [34]; Demoulin et al. [36]; Chapman et al. [42]; Hesselstrand et al. [23]; Hill [12]; Kallhed and Mårtensson [48]; Kurklinsky et al. [51]; Mathews [53]; Ravenek et al. [66]; Robinson et al. [19]; Robinson et al. [67]; Salgueiro et al. [69]; Stanos [71]; Stewart et al. [72]
Environmental modification	Artner et al. [31]; Bosy et al. [33]; Hesselstrand et al. [23]; Hill [12]; Kurklinsky et al. [51]; Paquette [59]; Silvestri [21]; Skjutar et al. [20]; Stewart et al. [72]; Ravenek et al. [66]; Von Bülow et al. [75]
*Focus on OCCUPATION* (task adaptation and occupation development)	
Pacing/graded activity	Bosy et al. [33]; Cammalleri [35]; Dobkin et al. [37]; Gonzalez et al. [43]; Hesselstrand et al. [23]; Hill [12]; Kallhed and Mårtensson [48]; Kurklinsky et al. [51]; McLean [55]; McLean et al. [56]; Nieuwenhuizen et al. [58]; Paquette [59]; Robinson et al. [19]; Robinson et al. [67]; Simon and Collins [70]; Stanos [71]; Stewart et al. [72]; van Huet et al. [74]
Activity (task) adaptation/therapeutically activity	Cammalleri [35]; Fisher et al. [13]; Gonzalez et al. [43]; Hill [12]; Jarrar [47]; Mathews [53]; McCormack and Gupta [54]; Nieuwenhuizen et al. [58]; Paquette [59]; Ravenek et al. [66]; Robinson et al. [19]; Rome [68]; Salgueiro et al. [69]; Simon and Collins [70]; Stewart et al. [72]; Von Bülow et al. [75]
Vocational intervention	Bosy et al. [33]; Caby et al. [80]; Demoulin et al. [36]; Gallice et al. [39]; Chapman et al. [42]; Harden et al. [44]; Harden et al. [45]; Hesselstrand et al. [23]; Hill [12]; Ravenek et al. [66]; Robinson et al. [19]; Salgueiro et al. [69]; Skjutar et al. [20]; van Huet et al. [74]
Sleep hygiene	Kallhed and Mårtensson [48]; Robinson et al. [19]; Simon and Collins [70]
Graded in vivo exposure	Nieuwenhuizen et al. [58]; Robinson et al. [19]
Yoga/Tai chi	Kallhed and Mårtensson [48]; McCormack and Gupta [54]
